# Evidence-Based TAM Classic Herbal Formula: From Myth to Science

**DOI:** 10.1155/2017/9493076

**Published:** 2017-01-26

**Authors:** Xingjiang Xiong, Chun-Tao Che, Francesca Borrelli, Kamal D. Moudgil, Giuseppe Caminiti

**Affiliations:** ^1^Department of Cardiology, Guang'anmen Hospital, China Academy of Chinese Medical Sciences, Beijing 100053, China; ^2^Department of Medicinal Chemistry and Pharmacognosy, College of Pharmacy, University of Illinois at Chicago, Chicago, IL 60612, USA; ^3^Department of Pharmacy, University of Naples Federico II, 80131 Naples, Italy; ^4^Department of Microbiology and Immunology, University of Maryland School of Medicine, 685 W. Baltimore St., HSF-1, Suite 380, Baltimore, MD 21201, USA; ^5^Cardiovascular Research Unit, Department of Medical Sciences, Centre for Clinical and Basic Research, IRCCS San Raffaele, Via della Pisana 235, 00163 Roma, Italy

In December 2011, 5 years ago, the* Nature* released a special issue on “Traditional Asian Medicine” (http://www.nature.com/nature/outlook/asian_medicine/) [1]. This issue marked a renaissance of interest in the field of complementary and alternative medicine. Three years later, the* Science* published its special issues on “The Art and Science of Traditional Medicine. Part 1: TCM Today—A Case for Integration; Part 2: Multidisciplinary Approaches for Studying Traditional Medicine; and Part 3: The Global Impact of Traditional Medicine.” This convergence is not occasional. From the new WHO traditional medicine strategy (2014–2023) to the application of systems biology in studying traditional medicine, a new clinical trend of integrating traditional medicine into modern health care has been gradually formed [2].

Traditional Asian medicine (TAM), a system of ancient medical practice including traditional Chinese medicine, Kampo, traditional Korean medicine, and Ayurveda, is widely used in Asian countries [3]. It is gaining increasing popularity in western countries [4]. TAM classic herbal formula, one of the important therapeutic modalities of TAM, has both unique theories and rich experience in the past thousands of years and is still being widely applied in modern clinic. It is defined as a formula that has been recorded in ancient classic medical books with fixed herbal drug composition, definite curative effect, and fewer adverse reactions for certain diseases.

From the past to the present, TAM classic herbal formula has made countless contribution to well-being and public illness (cardiovascular diseases, diabetes, etc.) [5]. It has provided new strategies for the treatment of many complex, refractory illnesses. Currently, more and more evidences showing the efficacy from clinic provide us further understanding of the biological functions and potential mechanisms of TAM formula, including Maxingshigan-Yinqiaosan for H1N1 influenza virus [6], Gegen qinlian decoction for type 2 diabetes mellitus [7], Liujunzi decoction for chronic dyspepsia [8], and Hemp Seed pill for functional constipation [9]. On the other hand, integrative medicine, which combined traditional medicine with conventional western medicine treatment, is not only an innovative medical model in clinical practice, but also the bridge for traditional medicine toward the medical sciences [10]. Previous studies have also identified that TAM classic herbal formula as adjunctive therapy could exhibit synergetic effect with each other [11–13]. Additionally, due to the limitations of “more investments, less drugs” challenge in drug discovery and development, scientists have turned their attention to the natural herbal medicines [14–16]. It is a current trend that TAM classic herbal formula has provided new promising drugs and candidates recently.

However, there are still numerous limitations in the current use and research of TAM classic herbal formula. Due to the difficulties in retrieving and learning original literatures in Chinese, lacking high level of evidence for clinical recommendation and the data of toxicology, adverse effects, potential herb-drug interaction, and pharmacological mechanism, quite a number of TAM classic herbal formulas are still recognized as a “mystery” to the science world. Accordingly, the role of these classic herbal formulas still needs more scientific and clinical data to verify their effectiveness and safety.

This special issue aims to summarize the current clinical and experimental progress of promising TAM classic herbal formulas and their active ingredients for various diseases. Altogether, 34 papers were gathered for publication, out of which 11 articles were accepted. The original research papers as well as comprehensive review articles on TAM classic herbal formula cover a wide range of topics, including clinical trial, systematic review, experimental study, and perspective.

One clinical trial addressed the clinical indications of two TAM classic herbal formulas including Danggui Shaoyao powder (tokishakuyakusan) and Guizhi Fuling pill (keishibukuryogan) for patients with dysmenorrhea. The paper titled “The difference between the Two representative Kampo Formulas for Treating Dysmenorrhea: An Observational Study” proposed models for predicting the use of these two formulas. The indications of Danggui Shaoyao powder included lightheadedness, BMI < 18.5, and a weak abdomen. The indications of Guizhi Fuling pill included tendency to sweat, heat intolerance, leg numbness, a cold sensation in the lower back, a strong abdomen, and paraumbilical tenderness and resistance. Four systematic reviews and meta-analyses summarized the efficacy and safety of some TAM classic herbal formulas, including Liuwei Dihuang pills for diabetic nephropathy, Huangqi jianzhong decoction for chronic gastritis, and other Chinese herbal formulas for breast cancer. Five experimental studies explored the pharmacological mechanism of TAM classic herbal formula and some active ingredients either in vivo or in vitro, including Sheng Mai powder on right ventricular dysfunction during chronic intermittent hypoxia, Yi Gong powder on iron homeostasis in acute inflammation, and other herbs and formulas for diabetes and tumor. Additionally, the role of* Nigella sativa* and its active constituents in learning and memory was also reviewed.

Recently, tremendous support and encouragement for research and development of TAM classic herbal formulas has been made either in policy or funding by the Chinese government and other Asian countries. Although skepticism and mysteriousness are still labeled by some physicians and patients, significant progress focusing on the effectiveness and safety of partial classic herbal formulas has been achieved. Unlike the classic pattern of “from bench to bedside” in translational medicine, it represents a new innovative research mode of “from bedside to bench to bedside.” This special issue presented the updated knowledge of partially used TAM classic herbal formulas. Accordingly, we hope that more evidence-based approaches on studying TAM classic herbal formula will be provided to lifting the mysterious veil.

*Xingjiang Xiong* *Chun-Tao Che* *Francesca Borrelli* *Kamal D. Moudgil* *Giuseppe Caminiti*
